# Immunoglobulin and T cell receptor repertoire changes induced by a prototype vaccine against Chagas disease in naïve rhesus macaques

**DOI:** 10.1186/s12929-024-01050-5

**Published:** 2024-06-01

**Authors:** Eric Dumonteil, Weihong Tu, Hans Desale, Kelly Goff, Preston Marx, Jaime Ortega-Lopez, Claudia Herrera

**Affiliations:** 1 Department of Tropical Medicine and Infectious Disease, School of Public Health and Tropical Medicine, and Vector-Borne and Infectious Disease Research Center, Tulane University, 1440 Canal St, New Orleans, Louisiana, 70112 USA; 2 Division of Microbiology, Tulane National Primate Research Center, Tulane University, Covington, LA USA; 3 Departamento de Biotecnología y Bioingeniería, Centro de Investigación y Estudios Avanzados del Instituto Politécnico Nacional, Ciudad de Mexico, México

**Keywords:** Trypanosoma cruzi, TCR, IgG, CDR3 domain, Immune response, RNA sequencing

## Abstract

**Background:**

A vaccine against *Trypanosoma cruzi*, the agent of Chagas disease, would be an excellent additional tool for disease control. A recombinant vaccine based on Tc24 and TSA1 parasite antigens was found to be safe and immunogenic in naïve macaques.

**Methods:**

We used RNA-sequencing and performed a transcriptomic analysis of PBMC responses to vaccination of naïve macaques after each vaccine dose, to shed light on the immunogenicity of this vaccine and guide the optimization of doses and formulation. We identified differentially expressed genes and pathways and characterized immunoglobulin and T cell receptor repertoires.

**Results:**

RNA-sequencing analysis indicated a clear transcriptomic response of PBMCs after three vaccine doses, with the up-regulation of several immune cell activation pathways and a broad non-polarized immune profile. Analysis of the IgG repertoire showed that it had a rapid turnover with novel IgGs produced following each vaccine dose, while the TCR repertoire presented several persisting clones that were expanded after each vaccine dose.

**Conclusions:**

These data suggest that three vaccine doses may be needed for optimum immunogenicity and support the further evaluation of the protective efficacy of this vaccine.

## Background

Chagas disease is a zoonotic parasitic disease of the Americas, caused by the protozoan parasite *Trypanosoma cruzi*, and transmitted primarily by hematophagous triatomine bugs. Disease burden reaches at least 6 millions cases in Latin America alone [1] and a global burden of 806,170 Disease-adjusted life years (DALYs) and an associated $627.46 million in health-care costs [2].

The disease starts with a short acute phase lasting a few weeks with flu-like signs and symptoms, during which *T. cruzi* parasites proliferate in the blood and a high parasitemia can be detected. This is followed by a chronic phase during which there are few circulating blood parasites as most replicate as intracellular amastigotes in cardiac, skeletal and smooth muscle cells. The chronic phase is initially asymptomatic, but 30–40% of infections will progress to symptomatic cardiac or digestive disease, sometimes decades after infection [3]. Only two drugs are available for treating patients, benznidazole and nifurtimox, but their efficacy decreases dramatically as disease progresses, and their side effects lead to frequent treatment interruptions and non-compliance [4–6]. Structural barriers for accessing diagnostic testing and treatment also considerably restrict access to appropriate care for patients [7–9].

Because of the limitations of current drug treatments, a vaccine would be an excellent additional tool for disease control. Many studies have shown the feasibility of multiple vaccine antigens and platforms in mice [10, 11], and a few vaccine candidates have been tested in other animal models including dogs and non-human primates. Among these, a therapeutic DNA vaccine based on Tc24 and TSA1 parasite antigens has been shown to be effective to prevent cardiac alterations caused by *T. cruzi* infection in experimentally-infected macaques [12]. Tc24 is calcium binding protein associated with the parasite flagellar pocket, and TSA1 is part of the trans sialidase family of proteins. A recombinant protein version of this vaccine has been developed [13–15], based on Tc24-C4 and TSA-C4 antigens which incorporate specific cysteine replacements to improve recombinant protein expression and purification from *Escherichia coli*, while maintaining the antigenicity of the proteins [16–18]. The subcutaneous immunization of naïve macaques with three doses of recombinant Tc24-C4 and TSA1-C4 antigens formulated with a TLR4 adjuvant was found to be safe and immunogenic [19]. Indeed, high titers of antibodies and antigen-specific cytokine production by T cells were detected following, warranting a more detailed evaluation of the immune response induced by vaccination.

Here we performed a transcriptomic analysis of PBMC responses to vaccination in naïve rhesus macaques, to shed light on the immunogenicity of this Chagas disease vaccine and guide the optimization of vaccine doses and formulation. In particular, we focus on identifying IgG and T cell receptor (TCR) repertoires, which can inform in detail on the breadth of the immune response induced by vaccination and the potential expansion of specific B and T cell clones [20–23]. Indeed, the antigen binding specificity of IgGs depend on the variable domains of the light and heavy chains, and more precisely their variable complementarity-determining region 3 (CDR3), which unique sequences result from recombination among the Ig variable (V), diversity (D) and joining (J) genes [24–26]. Similarly, TCR diversity relies on CDR3 regions within the alpha and beta subunits of the TCR, driven by recombination among TCR V/D/J genes [27, 28].

## Methods

### Animals and vaccination

Animals were housed at the Tulane National Primate Research Center (TNPRC) under the care of TNPRC veterinarians, in accordance with the standards incorporated in the Guide for the Care and Use of Laboratory Animals and with the approval of Tulane Institutional Animal Care and Use Committee (IACUC). Three naïve male rhesus macaques (*Macaca mulatta*) 4–5 years old were vaccinated with three doses of the vaccine based on Tc24-C4 and TSA1-C4 antigens formulated with a TLR4 adjuvant, one month apart, as described before [19]. A blood sample was collected in EDTA at baseline and one month after each vaccine dose. PBMCs were isolated and cryopreserved until used for RNA purification and RNA-sequencing.

### RNA purification and RNA sequencing

Cryopreserved PBMCs [19] were checked for viability with trypan blue, and about 10^6^ cells were used for RNA extraction using PerfectPure RNA Cultured Cell Kit™ (5 Prime, Inc.) following manufacturer instructions. RNA integrity was assessed on an Agilent BioAnalyzer and all samples had a RNA Integrity Number (RIN) > 8. About 200 ng of RNA was used for library preparation and sequencing on an Illumina MiSeq platform, and about 12–35 million reads/sample were obtained after quality filtering. Raw reads have been deposited in NCBI SRA database under Bioproject #PRJNA1010169, Biosamples SAMN37182435-SAMN37182446.

### Transcription profile

Reads were mapped to the Rhesus macaque reference genome (Mmul10 accession: GCF_003339765.1) in Geneious 11. Read counts were then normalized and differentially expressed genes called using DESeq2 as implemented in iDEP1.1 [29]. One sample was removed from further analysis (macaque KL72, second vaccine dose) due to a very low number of reads. Differences in gene expression levels > 1.5 fold change were called at a significance alpha of 0.05 adjusted for multiple testing using the false discovery rate (FDR) method. Volcano plots were used to visualize differentially expressed genes. Enriched functional pathway associated with up-regulated and down-regulated genes, respectively, were identified using ShinyGO 0.77 [30], based on Gene Ontology Biological Process database.

### Major histocompatibility complex typing

RNA-seq read counts mapping to major histocompatibility complex (MHC) reference genes (Mamu A, Mamu B, Mamu DPA, Mamu DPB, Mamu DQA, Mamu DQB and Mamu DRA) for individual macaques were used for de-novo assembly in Geneious. Assembled MHC sequences were compared with the IDP-MHC allele database [31] using BLAST and the top two matches with > 98% sequence identity for each MHC gene were retained.

### Antibody isotype

RNA-seq read counts mapping to unique 150 bp regions of immunoglobulin IgA, IgE, IgG1, IgG2, IgG3, IgG4 and IgM subclasses were used to assess the relative expression level of the respective antibody isotype flowing vaccination. These were normalized according to the total number of reads mapping to the macaque genome from each sample to account for differences in sequencing depth and coverage, and provide relative abundance level of Ig isotypes.

### IgG antibody repertoire

Reads mapping to the variable complementarity-determining region 3 (CDR3) region of IgG heavy chain gene, which mediates antibody specificity, as well as flanking regions were extracted for analysis of IgG variable domain repertoire. While using bulk RNA sequences may miss rare IgGs, as only a small fraction of total mRNA may be derived from IgG genes, it can nonetheless provide valuable information on the more abundant repertoire of IgGs [24–26]. Extracted CDR3 nucleotide sequences were analyzed using IgBLAST [32] to identify the combination of the variable (V) gene, diversity (D) gene and joining (J) gene and gene usage frequency for IgG production in individual macaques following vaccination. The translated CDR3 sequences were analyzed for frequency and similarity using the EFI Enzyme Similarity Tool [33] and similarity networks were elaborated in Cytoscape to visualize changes in antibody repertoire over time. CDR3 sequence diversity was further assessed using Shannon H and Richness S summary indices, which were calculated in Past4.0 [34].

### T cell receptor repertoire

Reads mapping to the CDR3 region of the T cell receptor (TCR) beta subunit gene, which mediates T cell epitope binding specificity, as well as flanking regions were extracted for analysis of TCR variable domain repertoire. As for the IgG repertoire, this approach is likely biased toward more abundant TCRs due to the low relative abundance of TCR mRNAs among samples, but it can identify key changes in TCR repertoire following vaccination. TCR variable domain sequences were analyzed using IMGT/HighV-QUEST [27, 28] to identify the combination of the TCR beta V/D/J genes from each sequence and TCR gene usage frequency and its changes following vaccination. The translated CDR3 sequences were analyzed for frequency, similarity and diversity as described above for the IgG repertoire.

### Statistical analysis

The statistical significance of differentially expressed genes, as well as that of enriched pathways was adjusted using the false discovery rate (FDR) method. The expression levels of individual cytokines and the proportion of Ig subclass were compared among the different time points using one way analysis of variance (ANOVA). Changes in the overall cytokine and Ig subclass profiles were assessed by linear discriminant analysis (LDA).

## Results

### PBMC transcriptomic profile following vaccination

**Fig. 1:**
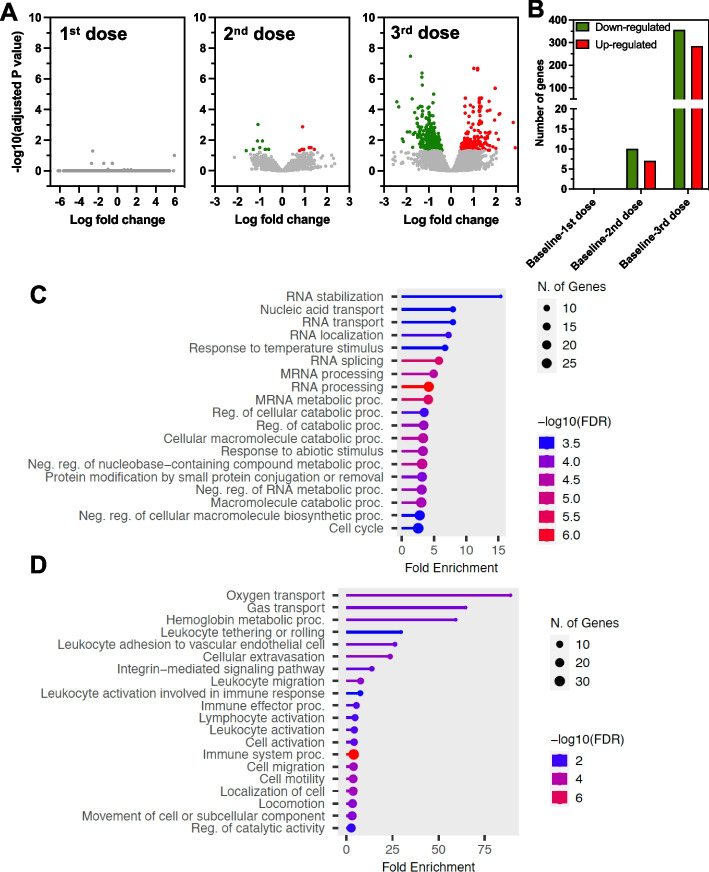
PBMC transcriptomic profile after vaccination. **A** Volcano plots of gene expression levels after each vaccine dose compared to baseline expression levels. Gray dots indicate genes with no significant difference in expression levels, while colored dots indicate genes that are significantly up- (Red) and down- (Green) regulated compared to baseline expression level. **B** Counts of up- (Red) and down- (Green) regulated genes after each vaccine dose (Adjusted *P* value < 0.05). Top 20 functional pathways associated with down- **(C)** and up- **(D)** regulated genes after vaccination (3 doses) based on GO Biological Process database. Circle size is proportional to the number of genes from the respective pathways that are differentially expressed and FDR-adjusted *P* values are color-coded as indicated

We first assessed the transcriptomic profile of PBMCs from vaccinated macaques one month after each of three vaccine doses, to further assess the immunogenicity of this candidate vaccine against *T. cruzi*. RNA-sequencing yielded 12–35 million quality reads/sample and over 80% were successfully mapped to the macaque genome. Differential expression analysis indicated that the gene expression profile of PBMCs was not significantly altered after the first vaccine dose, and only 17 genes were differentially expressed compared to baseline levels after the second vaccine dose. On the other hand, after the third vaccine dose, a total of 639 genes were differentially expressed, with 283 that were up-regulated and 356 down-regulated (Fig. 1A and B).

Pathway analysis was performed based on differentially expressed genes after the third vaccine dose. Biological pathways associated down-regulated genes following vaccination included multiple RNA processing pathways as well as several metabolic/catabolic pathways (Fig. 1C). On the other hand, up-regulated genes were associated with leukocytes and lymphocytes activation pathways and other immune system activation, as well as cell migration, adhesion or activation (Fig. 1D). These data suggested a strong activation of PBMCs and of the immune response after the three vaccine doses.

**Fig. 2:**
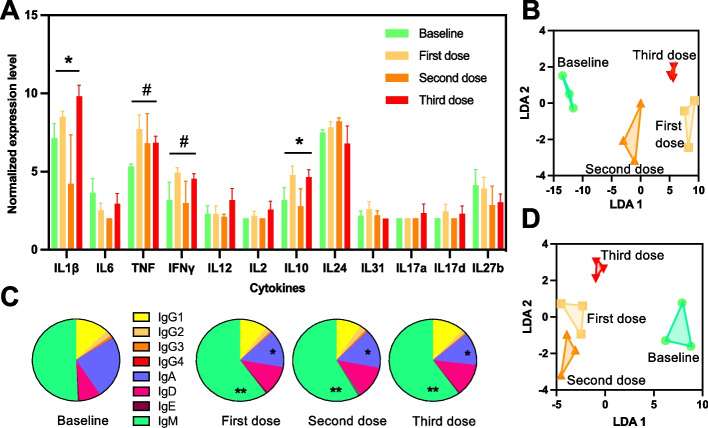
Cytokine and antibody expression profile induced by vaccination. **A** Cytokine expression profile is shown after each vaccine dose. Data are presented as mean ± SEM for the indicated cytokines (in arbitrary units), which cover some of the Th1, Th2, and Th17 cytokines. * indicates significant differences among groups (ANOVA, *P* < 0.05) and # nearly significant differences (ANOVA, *P* < 0.1). **B** LDA of cytokine expression profile indicating differences in profiles after each vaccine dose. **C** Ig subclass proportion after each vaccine dose. The subclasses are color coded as indicated. * and ** indicate significant differences with baseline level for the indicated Ig subclass (ANOVA, *P* < 0.05 and *P* < 0.01, respectively). **D** LDA of Ig subclass profile indicating differences in profiles after each vaccine dose

For a better understanding of the orientation of the immune response and its potential polarization, we assessed the changes in cytokine expression profile from the PBMCs. As shown in Fig. 2A, changes were observed in the cytokine profiles after each vaccine dose, with significant changes in IL1β and IL10, while TNF and IFNγ almost reached significance, and overall changes in cytokine profiles after each vaccine dose as indicated by LDA (Fig. 2B). However, no marked polarization was detected as Th1, Th2 and Th17 cytokines genes remained evenly expressed following vaccination. Analysis of Ig subclass expression profiles indicated limited subclass switch following the first vaccine dose, and no major changes after subsequent doses (Fig. 2C). Indeed, the first vaccine dose induced a significant increase in the proportion of IgM, mostly at the expense of IgA expression, which was reduced, and this pattern was maintained after subsequent vaccine doses. LDA analysis also indicated changes in Ig profiles after vaccination (Fig. 2D). Importantly, high levels of expression of IgG1 were sustained following vaccination, while no IgE was induced. Together, these results agree with the induction of a broad and non-polarized immune response following vaccination, and three vaccine doses appear to be needed for a strong transcriptomic response of PBMCs.

We then typed the MHC of the vaccinated macaques to assess potential MHC restriction of vaccine response. MHC class I alleles were the most diverse among the three macaques, with a total of 6 and 5 alleles identified for Mamu A and Mamu B genes, respectively, while MHC class II genes were less diverse, with 1–4 alleles among these macaques (Table 1). As all three macaques developed an immune response to the vaccine antigens, these were adequately processed through these diverse MHCs.

**Table 1:** MHC typing of vaccinated macaques

Macaque ID	KL72		LB83		LD53	
	Allele 1	Allele 2	Allele 1	Allele 2	Allele 1	Allele 2
Mamu A	A3*13	A1*008	A6:01	A1*003	A4*14	A1*004
Mamu B	B*052	B*072	B*060	B*024	B*002	B*052
Mamu DPA	DPA1*04	DPA1*04	DPA1*04	DPA1*04	DPA1*02	DPA1*02
Mamu DPB	DPB1*02	DPB1*02	DPB1*02	DPB1*03	DPB1*07	DPB1*15
Mamu DQA	DQA1*24	DQA1*26	DQA1*24	DQA1*24	DQA1*23	DQA1*26
Mamu DQB	DQB1*15	DQB1*18	DQB1*15	DQB1*18	DQB1*18	DQB1*18
Mamu DRA	DRA*01	DRA*01	DRA*01	DRA*01	DRA*01	DRA*01

### Changes in immunoglobulin G repertoire following vaccination

**Fig. 3:**
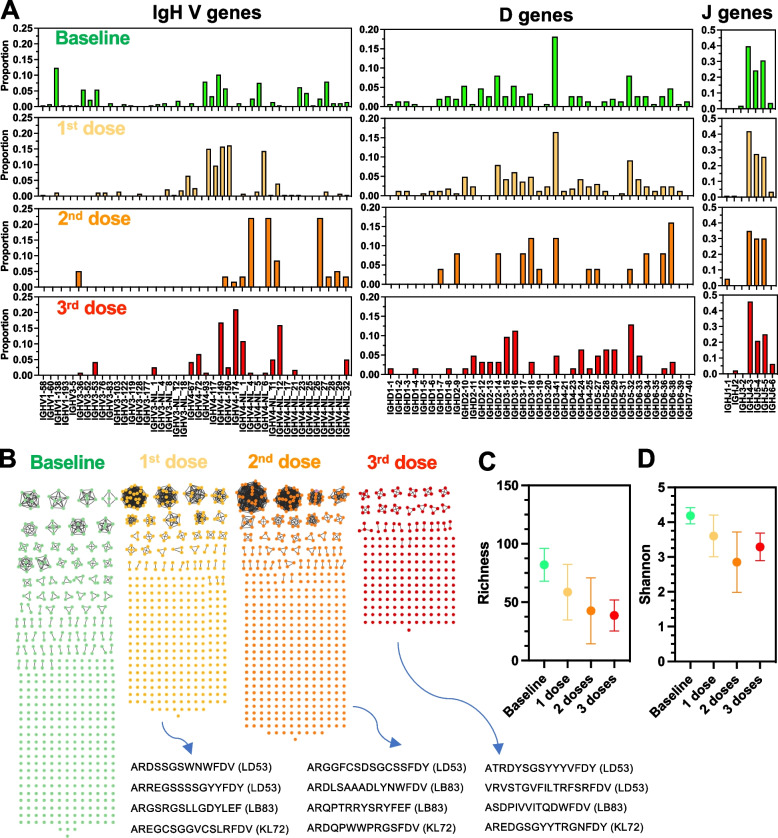
IgG repertoire following vaccination. **A** IgGH VDJ gene usage frequency after vaccination of macaque KL72. **B** Network analysis of IgG CDR3 domains following each vaccine dose. Each circle node represents a unique CDR3 sequence, and edges link identical sequences. Color codes of the CDR3 nodes indicate sequences present at baseline and after each vaccine dose, respectively. Note that no CDR3 sequence persisted overtime, and that new sequences were present at each time-point. In each macaque, specific CDR3 sequences were particularly enriched after each vaccine dose, likely representing expansion of antigen-specific sequences (Bottom sequences with macaque ID indicated in parenthesis). **C** Richness S and (**D**) Shannon H diversity indices of CDR3 sequences over time and following vaccination (*N* = 3 macaques)

IgG diversity results from random combinations of V, D and J gene segments [35, 36], to generate unique CDR3 within the hypervariable region of the protein which is involved in antigen-binding specificity [37]. Thus, we assessed the changes in IgG repertoire and Ig heavy chain VDJ gene usage following vaccination. A total of 2191 reads covering the IgG CDR3 region were obtained and resulted in 1756 productive sequences (80% of reads) for the identification of VDJ gene usage, corresponding to an average of 146 sequences per macaque and time point. As shown in Fig. 3A, changes in V and D gene usage could be detected after each vaccine dose, while J gene usage was mostly unaffected by vaccination. For example, IGHV1 and IGHD3 were the most frequently expressed genes prior to vaccination in macaque KL72, while after three vaccine doses IGHV4 gene was the most frequently used, as well as IGHD3 and IGHD5 genes (Fig. 3A). Similar changes were detected in the other macaques. These data suggested that different IgGs are produced after each vaccine doses. Therefore, we analyzed Ig CDR3 domain sequence diversity and elaborated networks illustrating antibody repertoire of all three vaccinated macaques. At baseline, an extensive diversity of CDR3 sequences were observed, but most were unique and only a few were present in more than one copy (Fig. 3B). Following the first vaccine dose, a dramatic reduction in CDR3 sequence diversity was observed, while several sequences were much more abundant, likely indicative of the clonal expansion of vaccine antigen-specific antibodies (Fig. 3B). For example, CDR3 sequences ARDSSGSWNWFDV, ARREGSSSSGYYFDY, ARGSRGSLLGDYLEF, AREGCSGGVCSLRFDV expanded after the first vaccine dose. Interestingly, no CDR3 sequence detected at baseline persisted after the first vaccine dose, and all CDR3 sequences were novel, in agreement with the changes in VD gene usage detected above in all macaques. After the second vaccine dose, all CDR3 sequences induced by the first vaccine dose were replaced by novel CDR3 sequences, indicating a major turnover of antibody-producing cell clones. CDR3 diversity remained limited compared to the baseline level, and new CDR3 sequences were further expanded. These included ARGGFCSDSGCSSFDY, ARDLSAAADLYNWFDV, ARQPTRRYSRYFEF, ARDQPWWPRGSFDV. This turn-over process appeared to be repeated after the third vaccine dose as well, and a new repertoire of CDR3 sequences was detected, although with more limited clonal expansion of CDR3 sequences (Fig. 3B). Analysis of Richness (Fig. 3C) and Shannon (Fig. 3D) diversity indices over time confirmed that antibody diversity was reduced after the first and second vaccine dose, likely associated with the clonal expansion of a few vaccine antigen-specific IgGs, which may target different epitopes of the antigens after each vaccine dose.

### Changes in T cell receptor repertoire following vaccination

**Fig. 4:**
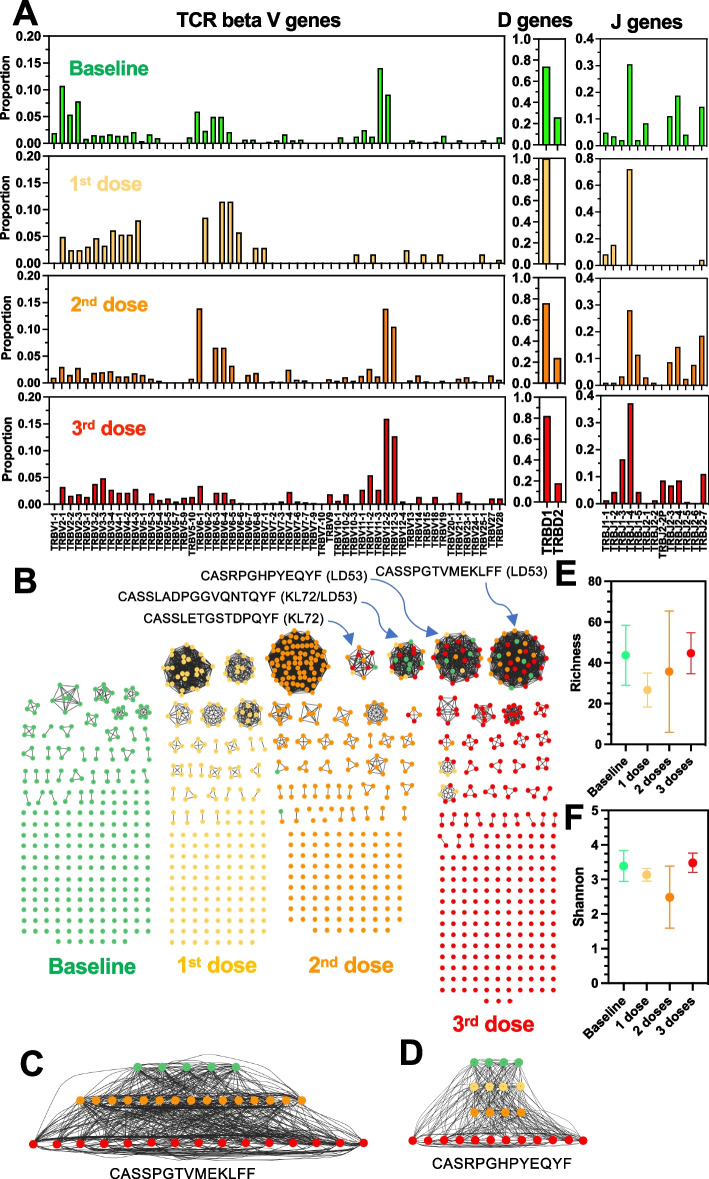
TCR beta repertoire following vaccination. **A** TCR beta VDJ gene usage frequency after vaccination of macaque LD53. **B** Network analysis of TCR beta CDR3 domains following each vaccine dose. Each circle node represents a unique CDR3 sequence, and edges link identical sequences. Color codes of the CDR3 nodes indicate sequences present at baseline and after each vaccine dose, respectively. Note that in addition of the identification of novel CDR3 sequences at each time-point following vaccination, some sequences present at baseline persisted and were enriched after each vaccine dose, likely representing clonal expansion of antigen-specific TCR sequences (Sequences indicated on top with macaque ID in parenthesis). **C** and (**D**) Magnified details of TCR beta CDR3 sequence networks illustrating their expansion after vaccination.** E** Richness S and (**F**) Shannon H diversity indices of CDR3 sequences over time and following vaccination (*N* = 3 macaques)

TCRs are responsible for detecting epitopes presented by MHC molecules and their diversity is also driven by rearrangements of TCR beta V, D and J gene fragments and TCR alpha V and J genes, to generate unique CDR3 regions that determine TCR epitope binding affinity and specificity [38, 39]. Thus, TCR beta CDR3 diversity and VDJ gene usage were similarly analyzed. A total of 4662 reads covering the TCR CDR3 region were obtained and resulted in 2965 productive sequences (66% of reads) for the identification of gene usage. TRB VDJ gene usage resulted altered starting with the first vaccine dose, particularly for V and J genes (Fig. 4A). For example, TRBV11 and TRBV12 genes frequently used at baseline were replaced by TRBV4 and TRBV6 which became the most used genes after the first vaccine dose in macaque LD53. These data are consistent with the induction of antigen-specific TCRs following vaccination. Further changes in gene usage were observed after the second vaccine dose, but limited changes seemed to occur after the third vaccine dose and TRBV12 and TRBJ1 genes were the most frequently used after vaccination.

Network analysis of TCR beta CDR3 domain diversity revealed further changes in the repertoire following vaccination (Fig. 4B). Indeed, vaccination had limited effects on overall TCR beta CDR3 diversity, although it tended to decrease after the first and second dose of vaccine as assessed by Richness and Shannon indices (Fig. 4E and F). Nonetheless, novel CDR3 sequences were induced after each vaccine dose and several. were present at high frequencies, likely representing antigen-specific TCR sequences. Moore strikingly, a few sequences present a low frequency at baseline persisted and were present at increasing frequencies following each vaccine dose, including the third dose (Fig. 4B, C and D). These corresponded to CDR3 sequences CASSPGTVMEKLFF, CASRPGHPYEQYF, CASSLADPGGVQNTQYF, or CASSLETGSTDPQYF, for example. One of these CDR3 sequence was also identified in two macaques (CASSLADPGGVQNTQYF, detected in macaques KL72 and LD53), indicating convergence of their immune response. These data evidenced a strong T cell response to vaccination, with the likely proliferation of cells with antigen-specific TCR beta.

## Discussion

The development of a Chagas disease vaccine would be a key step towards a better control of this neglected disease, and a vaccine prototype based on Tc24 and TSA1 antigens is emerging as an attractive candidate for further development [10, 13]. An initial evaluation of this vaccine in naïve macaque suggested that it could stimulate both B and T cell immunity [19]. We aimed here to expand these observations by assessing the transcriptomic response of PBMCs from vaccinated macaques and assess changes in their IgG and TCR repertoires. Indeed, the breadth and diversity of the IgG and TCR repertoires induced are important parameters of the immunogenicity of this vaccine, that can complement the identification of specific T cell subsets and other aspects of the immune response.

Transcriptomic analysis revealed that no changes in gene expression profiles were detected one month after the first vaccine dose, and only marginal changes one month after the second dose, so that three vaccine doses were required to detect significant alterations in the PBMC gene expression profile. This was somewhat unexpected as a large increase in antigen-specific IgG could already be detected one month after the first vaccine dose, indicating immunogenicity at this early time point [19]. However, the response to vaccines is largely asynchronous in humans, with early and delayed responses depending on the vaccine and its formulation [40]. Similarly, the transcriptomic response of PBMCs from macaques vaccinated with BCG is more important on day two after vaccination and decreases in the following weeks [41]. On the other hand, PBMCs from humans vaccinated with *Plasmodium falciparum* sporozoites present a transcriptomic response at day 27 post-immunization but not before [42]. Thus, the inclusion of additional time points would be needed for a fine-scale analysis of the kinetics of transcriptomic changes to this Chagas disease vaccine, as it is unclear what changes may occur in the few days after immunization. Also, increasing the sample size would allow fora greater power to identify differentially expressed genes as we observed many genes presenting potential changes in expression levels after the first vaccine dose, but none reached statistical significance.

On the other hand, there was a clear transcriptomic response of PBMCs after the third vaccine dose, with over 600 differentially expressed genes, confirming vaccine immunogenicity. These genes were involved in several metabolic/catabolic functions which appeared down regulated, suggesting changes in cell activity/differentiation. Also, as expected, several pathways associated with the activation of immune cells from leukocytes to T cells were upregulated, indicative of an active response of these immune cells. The immunoglobulin expression profile indicated limited subclass switch, as the main change was an increase in the proportion of IgM while IgA was reduced, and the other subclasses remained unaltered. In particular, the production of IgG1 remained predominant among IgGs, and this isotype is associated with the highest effector function activity in macaques [43]. Notably, the expression of IgE, which is related to allergic reactions [44] was negligible, suggesting a lack of allergic reaction to the vaccine. However, the analysis of nasal secretions would be required for confirmation [45]. Importantly, only the proportion of the Ig subclass was analyzed here, which does not preclude additional changes in total Ig levels.

Analysis of the cytokine expression profile also suggested the induction of a balanced immune response, including Th1, Th2 and Th17 cytokines. Such a balanced immune response would be indicated for an effective vaccine against *T. cruzi*, as a cellular response is critical for parasite control but hyperpolarization may lead to tissue damage [10, 46]. These data support the further evaluation of vaccine efficacy against *T. cruzi* infection.

Analysis of the IgG heavy chain repertoire indicated changes in their VDJ gene usage one month after the first vaccine dose, and after each following vaccine dose. These changes were associated with a renewal of the CDR3 repertoire after each vaccine dose, and no clone persisted over time, although some clones appeared to transiently expand. While our approach based on bulk RNA-sequencing may lack sensitivity to detect rare IgGs, it can nonetheless identify the most abundant antibodies. Rapid changes in Ig CDR3 repertoire have similarly been observed at 7 and 28 days post-vaccination with a pneumococcal vaccine in humans [47]. Also, the longitudinal follow-up of the human IgG repertoire showed that > 85% of CDR3s can be detected only once out of 24 time points over a 11 months period [48], indicating a very rapid turnover. Although vaccine antigens Tc24 and TSA1 are both highly conserved among *T. cruzi* strains [49, 50], the generation of diverse polyclonal antibodies against them after each vaccine dose may help broaden their binding affinity to accommodate for more sequence variants and/or increase their efficacy by targeting different epitopes of the antigens.

Vaccination also induced important changes in T cell receptor repertoire, with the notable persistence and expansion of specific CDR3 sequences over at least 4 months, and some convergence in two vaccinated macaques. While our approach may also be biased towards more abundant TCRs, it is even more remarkable that several TCRs persisted over time at increased frequency. These expanded CDR3 clones are likely antigen specific and further studies should help assess this specificity. These data evidence a clear T cell response to vaccination and agree with the activation of T cells targeting multiple epitopes from the vaccine antigens, and their expansion over at least 4 months after the first vaccine dose suggest the presence of memory T cells. Vaccination in humans has often been associated with increases in TCR beta CDR3 diversity, for example in response to a Rabies virus vaccine [51] or a Hepatitis B vaccine [52]. Still, our data suggest a more focused response to our vaccine candidate with limited expansion of the TCR repertoire. This may be due to differences among vaccine antigens and their immune processing or differences in the kinetics of these responses, as more time points would be needed for a more detailed analysis of their time course, as mentioned above. Also, while two vaccine doses were sufficient to induce changes in TCR repertoire and expansion of some CDR3 clones, the third vaccine dose brought further expansion of these clones and may thus be needed for a stronger immunogenicity of this vaccine. Future studies should help assess potential differences in protective efficacy after two or three vaccine doses.

Remarkably, all three vaccinated macaques presented transcriptomic responses and changes in their IgG and TCR repertoires following vaccination, in the context of a varied MHC background, particularly for class I MHC. This is also encouraging as it suggests that so far there are no MHC restrictions of vaccine immunogenicity [53, 54] and future studies should include animals with additional MHC background to broaden this evaluation.

## Conclusion

We identified here a clear transcriptomic response of PBMCs from macaques vaccinated with a Chagas disease vaccine based on Tc24 and TSA1 parasite antigens, with the up-regulation of several immune cell activation pathways. These data confirming the immunogenicity of this vaccine, with a broad non-polarized immune profile. While changes in IgG and TCR repertoires could be detected one month after the first vaccine dose, further changes were observed after the second and thirddoses. The IgG repertoire showed a rapid turnover with new IgGs following each vaccine dose, while the TCR repertoire presented persisting clones that were expanded after each vaccine dose, suggesting that three vaccine doses may be needed for optimum efficacy. This work warrants the further characterization of the IgG and T cell receptor repertoires of Chagas disease patients [55], particularly with different stages of disease progression/parasite control, to identify immune repertoire profiles associated with better disease outcomes and correlates for protection. Also, evaluating the protective efficacy of this vaccine candidate against *T. cruzi* infection will be key for vaccine development and the availability of naturally-infected macaques provide an ideal model for testing a therapeutic vaccine [56].

## Data Availability

The datasets generated and/or analyzed during the current study are available in the NCBI SRA database under Bioproject #PRJNA1010169, Biosamples SAMN37182435-SAMN37182446.
